# Supplementary material for: Binding and Action of Triphenylphosphonium Analog of Chloramphenicol upon the Bacterial Ribosome
Source: Antibiotics (Basel). 2021 Apr 5;10(4):390. doi: 10.3390/antibiotics10040390 (PMC8066774; doi:10.3390/antibiotics10040390)
Supplement: Supplementary file 1 [file antibiotics-10-00390-s001.pdf]

Supplementary Information for

Binding and action of triphenylphosphonium analog of chloramphenicol upon the bacterial ribosome.

Chih-Wei Chen[1,*], Julia A. Pavlova[2,*], Dmitrii A. Lukianov[3], Andrey G. Tereshchenkov[4], Gennady I. Makarov[5], Zimfira Z. Khairullina[2], Vadim N. Tashlitsky[2], Alena Paleskava[6,7], Andrey L. Konevega[6,7,8], Alexey A. Bogdanov[2,4], Ilya A. Osterman[2,3,#], Natalia V. Sumbatyan[2,4,#] and Yury S. Polikanov[1,9,10,#]

[1] Department of Biological Sciences, University of Illinois at Chicago, Chicago, IL 60607, USA
[2] Department of Chemistry, Lomonosov Moscow State University, Moscow, 119992, Russia
[3] Center of Life Sciences, Skolkovo Institute of Science and Technology, Skolkovo, 143028 Russia
[4] A.N. Belozersky Institute of Physico-Chemical Biology, Lomonosov Moscow State University, Moscow, 119992, Russia
[5] Laboratory of Multiscale Modeling of Multicomponent Materials, South Ural State University, Chelyabinsk, 454080, Russia
[6] Petersburg Nuclear Physics Institute, NRC “Kurchatov Institute”, Gatchina, 188300, Russia
[7] Peter the Great St.Petersburg Polytechnic University, Saint Petersburg, 195251, Russia
[8] NRC “Kurchatov Institute”, Moscow, 123182, Russia
[9] Department of Pharmaceutical Sciences, University of Illinois at Chicago, Chicago, IL 60607, USA
[10] Center for Biomolecular Sciences, University of Illinois at Chicago, Chicago, IL 60607, USA

[*] These authors contributed equally to this work: Chih-Wei Chen, Julia A. Pavlova;
[#] Correspondence: i.osterman@skoltech.ru (I.A.O.)
sumbtyan@belozersky.msu.ru (N.V.S.)
yuryp@uic.edu (Y.S.P.)

This file includes:

I. Supplementary Methods;
II. Supplementary Table 1;
III. Supplementary Figures 1 through 4 with legends;
IV. Supplementary References;

I. SUPPLEMENTARY METHODS

Chemical synthesis.

The general scheme for the synthesis of CAM-C4-TPP is shown in **Figure 1**. Chloramphenicol amine (CAM, **2**, (1R,2R)-2-amino-1-(4-nitrophenyl)propane-1,3-diol) was prepared via acid hydrolysis of chloramphenicol (CHL, **1**) according to the previously published procedure [1], which was also used in our recent studies [2,3]. (4-Carboxybutyl)triphenyl-phosphonium bromide (C4-TPP, **5**) was obtained by condensation of 5-bromopentanoic acid (**3**) and triphenylphosphin (**4**) for 12 hours at 85°C, and then activated by reaction with 1-hydroxysuccinimide in the presence of N,N'-dicyclohexylcarbodiimide at 0°C. The resulting succinimide-reactive ester was used for the acylation of CAM in the presence of diisopropylethylamine as a base at room temperature. The resulting CAM-C4-TPP compound was purified by column chromatography on silica gel. The purity and chemical structure of the obtained compound was confirmed by HPLC, LC-MS, and NMR spectroscopy (see below).

*(4-Carboxybutyl)triphenylphosphonium bromide (**5**).* Triphenylphosphin (1.0 g, 3.8 mmol) was condensed with 5-bromopentanoic acid (688 mg, 3.8 mmol) during 12 h at 85°C. The product was purified on silica gel 60 using the chloroform – methanol mixture, 4:1 (v/v) as eluent to give the pure product (**5**) with 90% yield (1.51 g). TLC: R_f($CHCl_3$: MeOH, 4:1) 0.30. LC-MS m/z calculated for $C_{23}H_{24}O_2P$ $(M)^+$ 363.2, found 363.2; t_R = 0.78 min. ^{1}H NMR ($CDCl_3$, 400 MHz) δ (ppm) 7.81-7.61 (15H, m, Ph), 3.60-3.48 (2H, m, -C<u>H</u>$_2$-P$^+$Ph$_3$), 2.49 (2H, t, J = 7.0 Hz, -C<u>H</u>$_2$-COOH), 1.87 (2H, p, J = 7.1 Hz, -C<u>H</u>$_2$-CH$_2$-COOH), 1.68 (2H, sextet, J = 7.7 Hz, -C<u>H</u>$_2$-CH$_2$-P$^+$Ph$_3$). ^{13}C NMR ($CDCl_3$, 101 MHz,) δ (ppm) 174.96 (-COOH), 135.24 (d, $J_{C,P}$ = 3.0 Hz, Ph$_{para}$), 133.67 (d, $J_{C,P}$ = 10.1 Hz, Ph$_{ortho}$), 130.68 (d, $J_{C,P}$ = 12.4 Hz, Ph$_{meta}$), 118.09 (d, $J_{C,P}$ = 85.9 Hz, Ph$_{ipso}$), 33.75 (-<u>C</u>H$_2$-COOH), 25.61 (d, $J_{C,P}$ = 16.9 Hz, -<u>C</u>H$_2$-CH$_2$-COOH), 22.39 (d, $J_{C,P}$ = 51.2 Hz, -<u>C</u>H$_2$-P$^+$Ph$_3$), 21.73 (d, $J_{C,P}$ = 4.2 Hz, -<u>C</u>H$_2$-CH$_2$-P$^+$Ph$_3$). ^{31}P NMR ($CDCl_3$, 162 MHz) δ (ppm) 24.00.

(5-[[(1R,2R)-1,3-dihydroxy-1-(4-nitrophenyl)propan-2-yl]amino]-5-oxopentyl)(triphenyl)phosphonium bromide (CAM-C4-TPP, 7). To the cold solution of 177 mg (0.40 mmol) of (4-carboxybutyl)triphenylphosphonium bromide (**5**) and 46 mg (0.40 mmol) of *N*-hydroxysuccinimide in 2 ml of anhydrous CH_2Cl_2 82 mg (0.40 mmol) of DCC was added at 0°C. The mixture was stirred for 2h at 0°C, then overnight at 4°C. The formed precipitate was filtered off, and the solvent was removed *in vacuo*. The residue was dissolved in 1 ml of DMF, then 100 mg (0.40 mmol) of chloramphenicol amine hydrochloride (**2**) and 140 µl (0.80 mmol) of DIPEA in 1 ml of DMF was added and the resulted mixture was stirred for 4h at RT. The reaction mixture was diluted with 20 ml of water and 1N aqueous HCl was

added dropwise to neutral pH. Then the mixture was extracted with $CHCl_3$ (3×15 ml), and the combined organic extracts were washed with 5% solution of $NaHCO_3$ (1×10 ml) and water (3×10 ml). Organic layer was dried over anhydrous Na_2SO_4, the volatiles were evaporated *in vacuo*. The target product was isolated from residue by purification on silica gel column eluting with solvents system $CHCl_3$: MeOH, 6:1. Yield: 190 mg (75%); TLC: R_f ($CHCl_3$: MeOH, 4:1) 0.58, R_f ($CHCl_3$: MeOH, 9:1) 0.18; LC-MS m/z calculated for $C_{32}H_{34}N_2O_5P$ $(M)^+$ 557.23, found 557.43; t_R = 1.33 min; ESI-MS m/z calculated for $C_{32}H_{34}N_2O_5P$ $(M)^+$ 557.23, found 557.22. ^1H NMR ($CDCl_3$, 400MHz) δ (ppm) 8.96 (1H, d, J = 7.4 Hz, -C(O)-N$\underline{H}$-), 8.03 (2H, d, J = 8.6 Hz, NO_2-Ph_{ortho}), 7.87-7.62 (15H, m, Ph), 7.59 (2H, d, J = 8.6 Hz, NO_2-Ph_{meta}), 5.03 (1H, d, J = 5.5 Hz, -C$\underline{H}$-OH), 4.07 (1H, ddt, J = 7.4, 5.5, 4.4 Hz, -NH-C$\underline{H}$-), 3.67 (1H, dd, J = 11.9, 4.4 Hz, -C$\underline{H}_2^a$-OH), 3.64-3.51 (2H, m, -C$\underline{H}_2$-P^+Ph_3), 3.49 (1H, dd, J = 11.9, 4.7 Hz, -C$\underline{H}_2^b$-OH), 2.59-2.43 (2H, m, -C$\underline{H}_2$-C(O)-NH-), 1.91-1.82 (2H, m, -C$\underline{H}_2$-CH_2-C(O)-NH-), 1.65 (2H, ddt, J = 19.7, 14.4, 6.7 Hz, -C$\underline{H}_2$-CH_2-P^+Ph_3). ^{13}C NMR ($CDCl_3$, 101 MHz) δ (ppm) 174.96 (C=O), 150.35 (NO_2-Ph_{ipso}), 147.08 (NO_2-Ph_{para}), 135.27 (3C, d, $J_{C,P}$ = 3.0 Hz, Ph_{para}), 133.67 (6C, d, $J_{C,P}$ = 10.1 Hz, Ph_{ortho}), 130.66 (6C, d, $J_{C,P}$ = 12.5 Hz, Ph_{meta}), 127.73 (2C, NO_2-Ph_{meta}), 123.21 (2C, NO_2-Ph_{ortho}), 118.12 (3C, d, $J_{C,P}$ = 86.0 Hz, Ph_{ipso}), 73.74 (-$\underline{C}$H-OH), 62.48 (-$\underline{C}H_2$-OH), 58.05 (-NH-$\underline{C}$H-), 33.99 (-$\underline{C}H_2$-C(O)-NH-), 26.48 (d, $J_{C,P}$ = 16.9 Hz, -$\underline{C}H_2$-CH_2-C(O)-NH-), 22.32 (d, $J_{C,P}$ = 51.1 Hz, -$\underline{C}H_2$-P^+Ph_3), 21.18 (d, $J_{C,P}$ = 4.0 Hz, -$\underline{C}H_2$-CH_2-P^+Ph_3). ^{31}P NMR ($CDCl_3$, 162 MHz) δ (ppm) 24.35.

N-[(1R,2R)-1,3-dihydroxy-1-(4-nitrophenyl)propan-2-yl]-5-(4,4-difluoro-5,7-dimethyl-4-bora-3a,4a-diaza-s-indacene-3-yl)pentanamide (BODIPY-CAM). To the solution of 0.2 mg (0.48 μmol) of N-hydroxysuccinimide ester of 4,4-difluoro-5,7-dimethyl-4-bora-3a,4a-diaza-s-indacene-3-pentanoic acid in 45 μl of DMF 0.2 mg (0.96 μmol) of chloramphenicol amine and 0.5 μl (2.9 μmol) DIPEA in 10 μl of DMF were added. The reaction mixture was stirred at room temperature for 3h, then overnight at 4°C. The resulting product was purified by HPLC (gradient of 20-100% MeCN in H_2O (0.01% TFA) for 30 min). Yield: 0.185 mg (75%); TLC: R_f ($CHCl_3$: MeOH, 9:1) 0.53; LC-MS m/z calculated for $C_{25}H_{29}BF_2N_4NaO_5$ $(M+Na)^+$ 537.2, found 536.8; t_R = 0.90 min; UV (MeOH): λ_{max} = 505, 275 nm; fluorescence (MeOH): λ_{ex} = 506 nm, λ_{em} = 512 nm.

Characterization of the synthesized CAM-C4-TPP compound.

Thin-layer chromatography (TLC) was carried out on silica gel 60 F254 plates (Merck); column chromatography was performed on silica gel 60 (0.063–0.2 mm) (Macherey Nagel). Spots were visualized by UV and Dragendorff's reagent.

Liquid chromatography coupled with mass spectrometry (LC-MS) was carried out using a UPLC/MS/MS system containing Acquity UPLC chromatography system (Waters, USA), Acquity BEH C18 column (2.1×50 mm, 1.7 µm bead size), and quadrupole mass-spectrometer TQD (Waters, USA) (electrospray ionization (ESI-MS) in the positive ion mode). Liquid chromatography was performed at 0.5 ml/min flow rate in 5-100% gradient of acetonitrile in the buffer containing 20 mM formic acid for 3 minutes.

Analytical and preparative reverse phase HPLC were performed on a Knauer semipreparative chromatograph (Germany) with a Beckman Coulter Ultrasphere ODS (10 × 250 mm, 5 µm) column in a gradient of MeCN in the aqueous solution of TFA (0.01%) with elution rate of 5 ml/min.

ESI-MS was done with Qtrap 3200 (AB Sciex, Canada) mass spectrometer. For the MS, the following conditions were used: ion spray voltage, 5500 V; ion source heater temperature, 350ºC; ion source gas (N_2) for nebulizing, 30 psi; ion source gas (N_2) for drying solvent, 40 psi; curtain gas (N_2), 11 psi.

NMR spectra were recorded using Agilent 400-MR (Agilent Technologies, USA) (400 MHz for ^{1}H, 101 MHz for ^{13}C and 162 MHz for ^{31}P) spectrometer. The chemical shift values are reported as δ ppm relative to TMS used as internal standard, and the coupling constants (J) are measured in Hz.

UV absorption spectra were recorded using Cary 50 Bio spectrophotometer (Varian, Australia). Fluorescence spectra were recorded on a Varian Cary Eclipse fluorescence spectrophotometer (Varian, Australia). Fluorescence anisotropy was measured with a VICTOR X5 Multilabel Plate Reader (Perkin Elmer, USA). The excitation wavelength was 485 nm, and the emission wavelength was 535 nm.

II. SUPPLEMENTARY TABLES

Table S1. X-ray data collection and refinement statistics.

Crystals		**70S ribosome in complex with Protein Y and CAM-C4-TPP**
Diffraction data		
Space Group		$P2_12_12_1$
Unit Cell Dimensions, Å (a x b x c)		209.50 x 448.64 x 621.27
Wavelength, Å		0.9795
Resolution range (outer shell), Å		311-2.80 (2.87-2.80)
$I/\sigma I$ (outer shell)		6.08 (0.86)
Resolution at which $I/\sigma I$=1, Å		2.80
Resolution at which $I/\sigma I$=2, Å		3.00
CC(1/2) at which $I/\sigma I$=1, %		18.7
CC(1/2) at which $I/\sigma I$=2, %		53.4
Completeness (outer shell), %		97.5 (97.1)
R_{merge} (outer shell)%		20.1 (150.7)
No. of crystals used		1
No. of Reflections Used:	Observed	4,696,602
	Unique	1,382,525
Redundancy (outer shell)		3.4 (3.3)
Refinement		
Resolution range of the diffraction data included in the refinement, Å		152-2.80
R_{work}/R_{free}, %		21.8/26.8
No. of Non-Hydrogen Atoms		
RNA		192,347
Protein		93,016
Ions (Mg, K, Zn, Fe)		2,582
Waters		9,105
Ramachandran Plot		
Favored regions, %		92.08
Allowed regions, %		6.99
Outliers, %		0.94
Deviations from ideal values (RMSD)		
Bond, Å		0.003
Angle, degrees		0.655
Chirality		0.037
Planarity		0.004
Dihedral, degrees		16.213
Average B-factor (overall), Å^2		53.9

III. SUPPLEMENTARY FIGURES

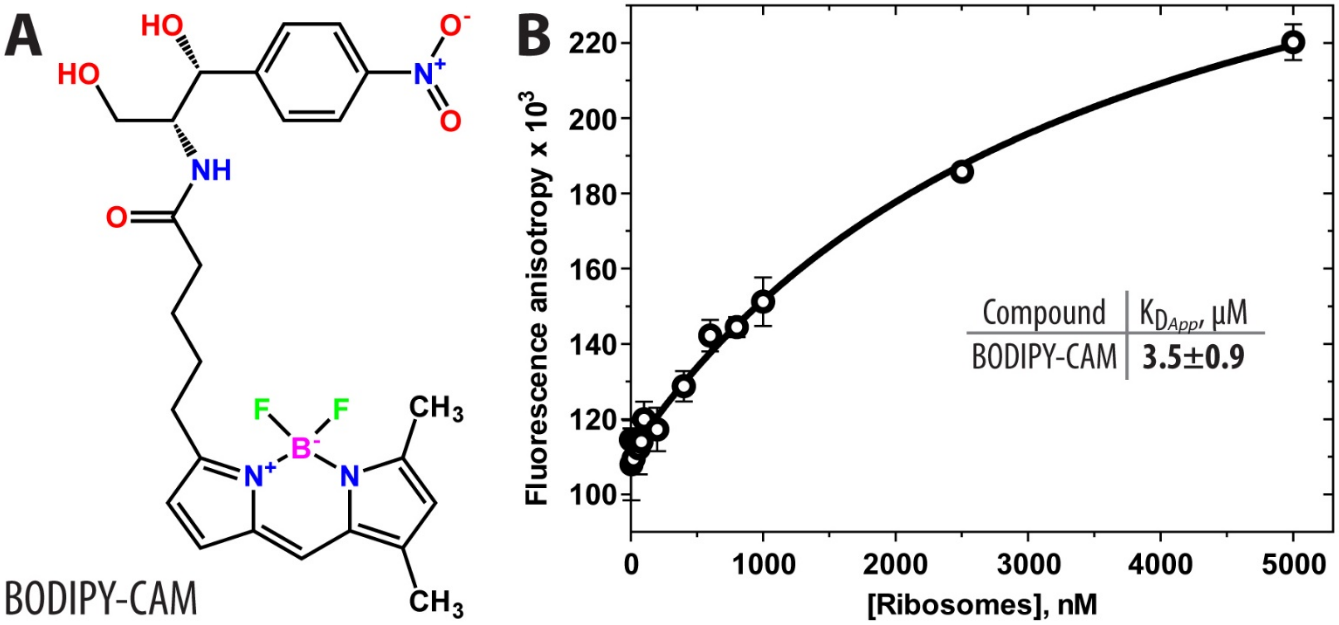

Figure S1. Binding of BODIPY-CAM to *E. coli* 70S ribosomes measured by fluorescence anisotropy. (**A**) Chemical structure of fluorescent analog of chloramphenicol BODIPY-CAM. (**B**) Equilibrium binding isotherm of BODIPY-CAM to *E. coli* 70S ribosomes. Non-linear regression analysis of obtained data yielded an apparent dissociation constant of BODIPY-CAM (K_{Dapp} = 3.5 ± 0.9 µM).

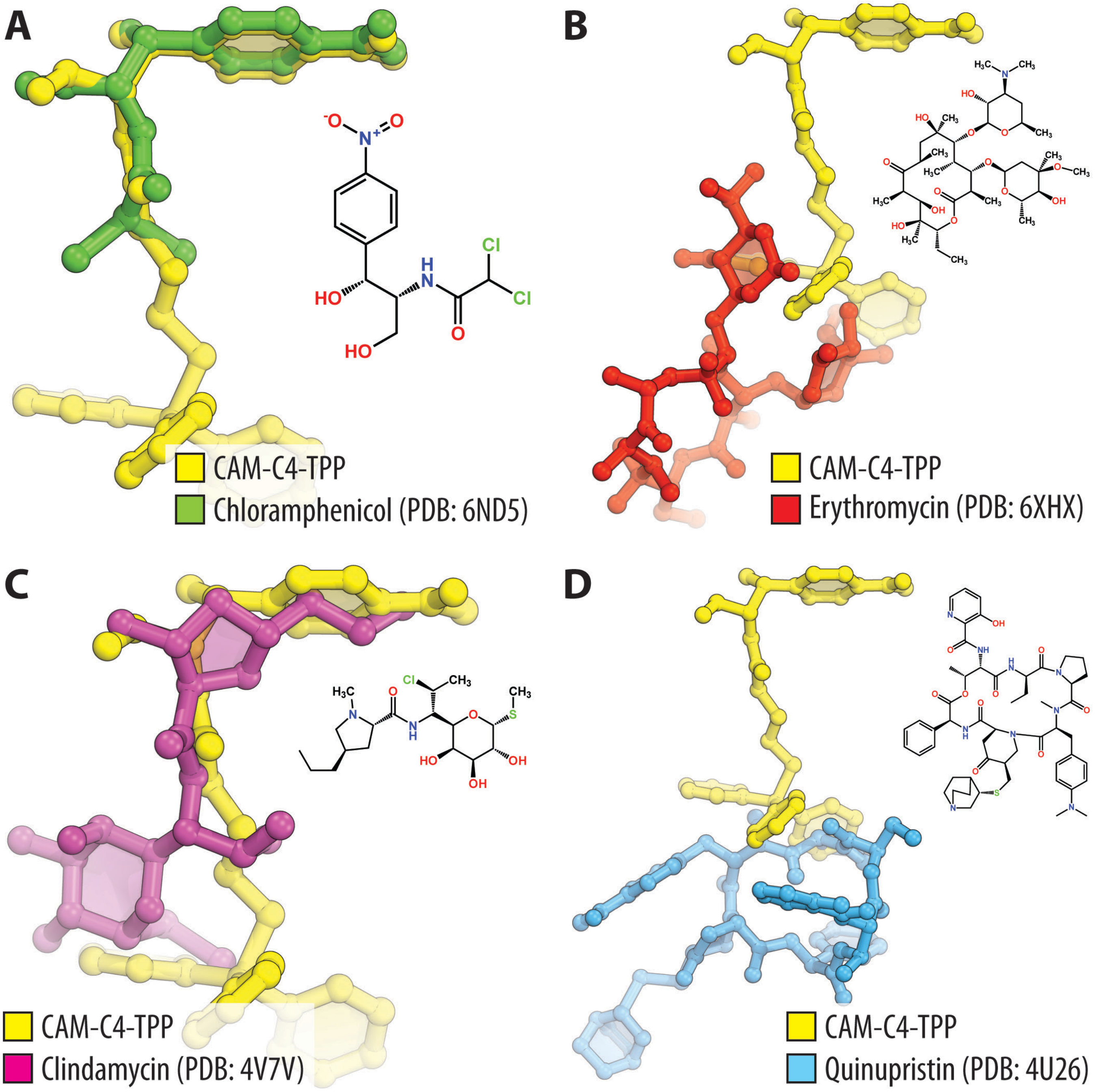

Figure S2. Superposition of CAM-C4-TPP with PTC-targeting CHL and several NPET-binding antibiotics. Superposition of the structures of the ribosome-bound CAM-C4-TPP (yellow) and (**A**) chloramphenicol (green, PDB entry 6ND5 [4]); (**B**) macrolide antibiotic erythromycin (red, PDB entry 6XHX [5]); (**C**) lincosamide antibiotic clindamycin (magenta, PDB entry 4V7V [6]); and (**D**) type B streptogramin quinupristin (blue, PDB entry 4U26 [7]). All structures of ribosome-bound antibiotics were aligned based on the domain V of the 23S rRNA.

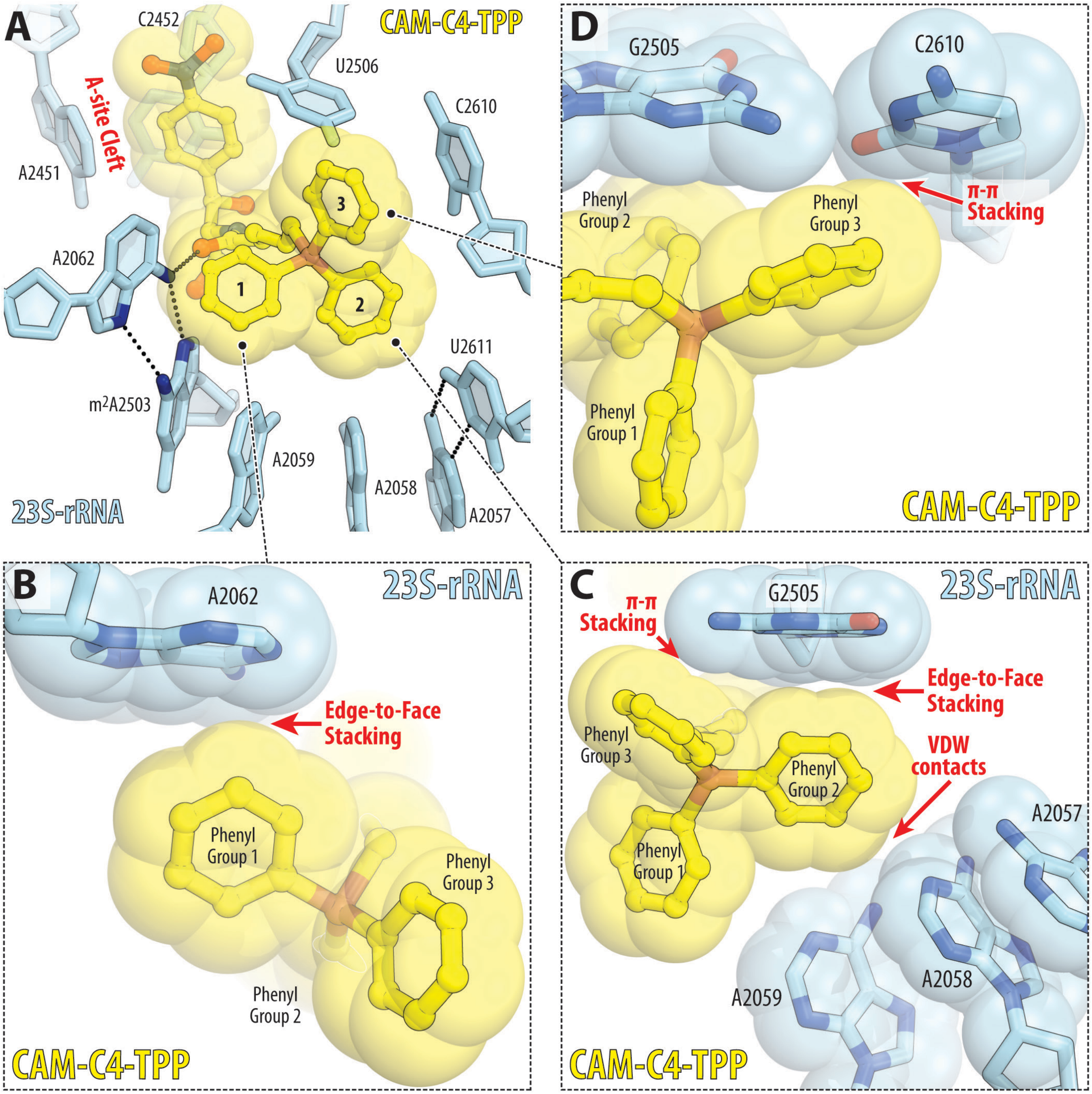

Figure S3. Hydrophobic interactions of the TPP moiety with the 23S rRNA nucleotides in the NPET. (**A**) Binding site of CAM-C4-TPP spanning the PTC and NPET of the 70S ribosome. This panel is identical to **Figure 4D** with phenyl groups of the TPP moiety arbitrary numbered 1, 2, and 3. (**B, C, D**) Close-up views of various hydrophobic Van der Waals interactions between the three phenyl groups of the TPP moiety and the 23S rRNA nucleotides lining up the ribosomal exit tunnel (highlighted with red arrows).

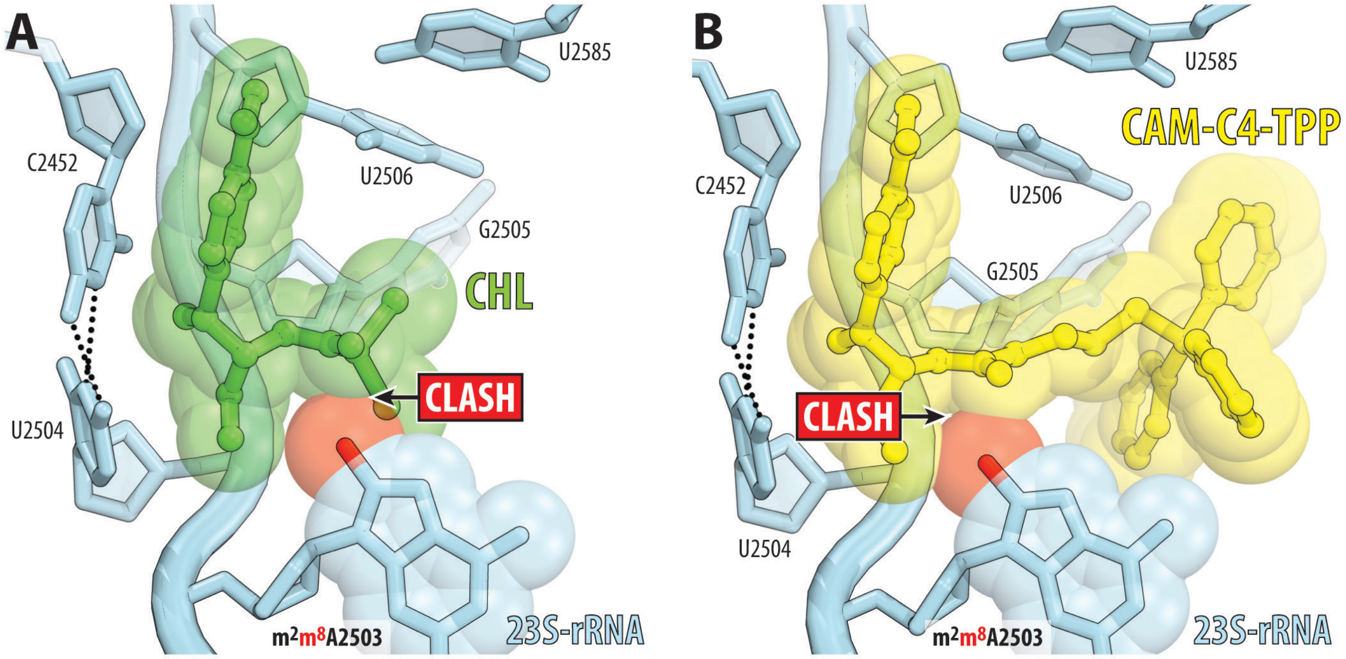

Figure S4. Structural basis for the Cfr-mediated resistance to CHL (A) and CAM-C4-TPP (B). Molecular modeling of the C8-methylation of A2503 (red sphere) catalyzed by the Cfr-methyltransferase reveals a small clash with both CHL (**A**) and CHL analog CAM-C4-TPP (**B**).

IV. SUPPLEMENTARY REFERENCES

1. Rebstock, M.C.; Crooks, H.M.; Controulis, J.; Bartz, Q.R. Chloramphenicol (chloromycetin). 1 iv. 1a chemical studies. *Journal of the American Chemical Society* **1949**, *71*, 2458-2462.

2. Tereshchenkov, A.G.; Shishkina, A.V.; Karpenko, V.V.; Chertkov, V.A.; Konevega, A.L.; Kasatsky, P.S.; Bogdanov, A.A.; Sumbatyan, N.V. New fluorescent macrolide derivatives for studying interactions of antibiotics and their analogs with the ribosomal exit tunnel. *Biochemistry (Mosc)* **2016**, *81*, 1163-1172.

3. Tereshchenkov, A.G.; Dobosz-Bartoszek, M.; Osterman, I.A.; Marks, J.; Sergeeva, V.A.; Kasatsky, P.; Komarova, E.S.; Stavrianidi, A.N.; Rodin, I.A.; Konevega, A.L., *et al.* Binding and action of amino acid analogs of chloramphenicol upon the bacterial ribosome. *J Mol Biol* **2018**, *430*, 842-852.

4. Svetlov, M.S.; Plessa, E.; Chen, C.W.; Bougas, A.; Krokidis, M.G.; Dinos, G.P.; Polikanov, Y.S. High-resolution crystal structures of ribosome-bound chloramphenicol and erythromycin provide the ultimate basis for their competition. *RNA* **2019**, *25*, 600-606.

5. Svetlov, M.S.; Syroegin, E.A.; Aleksandrova, E.V.; Atkinson, G.C.; Gregory, S.T.; Mankin, A.S.; Polikanov, Y.S. Structure of erm-modified 70s ribosome reveals the mechanism of macrolide resistance. *Nat. Chem. Biol.* **2021**, *17*, 412-420.

6. Dunkle, J.A.; Xiong, L.; Mankin, A.S.; Cate, J.H. Structures of the *Escherichia coli* ribosome with antibiotics bound near the peptidyl transferase center explain spectra of drug action. *Proc. Natl. Acad. Sci. USA* **2010**, *107*, 17152-17157.

7. Noeske, J.; Huang, J.; Olivier, N.B.; Giacobbe, R.A.; Zambrowski, M.; Cate, J.H. Synergy of streptogramin antibiotics occurs independently of their effects on translation. *Antimicrob. Agents Chemother.* **2014**, *58*, 5269-5279.
